# Comparing and combining methods that enhance liquid–gas mass transfer in a batch reactor: Ultrasonic degassing, aeration by gas bubbling, and liquid agitation^☆^

**DOI:** 10.1016/j.ultsonch.2025.107389

**Published:** 2025-05-16

**Authors:** W. Ludwig Kuhn, Jean-Yves Hihn, Bjørn Winther Solemslie, Ole Gunnar Dahlhaug

**Affiliations:** a Norwegian Institute for Nature Research, Water and Biodiversity, Postboks 5685 Torgarden, Trondheim 7485 Trøndelag, Norway; b Universit́e Marie et Louis Pasteur, CNRS UMR 6213 UTINAM, 30 avenue de l’Observatoire, Besançon 25010 Bourgogne-Franche-Comté, France; c Norwegian University of Science and Technology, Faculty of Engineering, Department of Energy and Process Engineering, Kolbjørn Hejes vei 1B, Trondheim 7012 Trøndelag, Norway

**Keywords:** Liquid-gas mass transfer, TDG supersaturation, Ultrasonic degassing, Mass transfer enhancement

## Abstract

The present study investigates methods to enhance liquid–gas mass transfer in total dissolved gas (TDG) supersaturated water. Using a two-part experimental setup, water is first supersaturated using air, followed by an evaluation of the influence of various operating parameters on the volumetric liquid–gas mass transfer coefficient, $k_{L}a$, in a liquid batch reactor. The dependence of this coefficient on sonication parameters is significant because of high partial pressure gradients with TDG supersaturation. The tested methods to enhance degassing are: ultrasonic degassing, aeration by gas bubbling, and agitation through flow circulation. The influence of the acoustic frequency and power, the gas diffuser porosity and flow rate, and the flow direction and velocity within the reactor were the parameters investigated for the respective method. In addition, a combination of the different methods was conducted to evaluate promoting effects on the liquid–gas mass transfer. Applying high-power ultrasound resulted in the largest mass transfer enhancement overall, even though aeration and liquid agitation increase the mass transfer when compared to natural degassing. Only a combination of ultrasound and liquid agitation through countercurrent flow leads to a small further enhancement in the mass transfer. Combining aeration by gas bubbling and ultrasound hampers the sonication process, but yields higher $k_{L}a$ values compared to aeration alone. The results indicate that high-power ultrasound, in combination with selected parameters, presents a powerful approach for efficient degassing of TDG supersaturated water. In addition, practical insights for applications within other fields are provided.

## Highlights

• Enhancement of liquid-gas mass transfer in total dissolved gas supersaturated water.
• Ultrasound, aeration, and liquid agitation tested; ultrasound yields highest increase.
• Significant effects of ultrasound due to high partial pressure gradients.
• Combining methods only gives a small further increase of liquid-gas mass transfer.

## 1 Introduction

The ongoing changing climate enforces a shift in the energy sector to low- or zero-emission energy sources [1]. In Norway, most of the electricity was historically produced by hydropower, due to its favorable topography and climate [2,3]. Nowadays, this monopoly is changing slightly, as the installed capacity of other renewable energy sources like wind and solar photovoltaic is growing. Nevertheless, the fleet of more than 1800 hydropower plants over all of Norway is still responsible for about 90 % of the electricity generation [4,5]. It is expected that, with a further increase in the capacity of intermittent energy sources, the purpose of hydropower within the energy mix will change from a pure base-load provider towards more flexibility [6,7].

In addition to being renewable and low in greenhouse gas emissions, hydropower plants serve a purpose in flood dampening [8], which is especially important for Norway, as climate models predict an increase in extreme precipitation events [9,10,11]. Despite bringing along these positive aspects, hydropower has several downsides, among which environmental harms play a vital role in its social acceptance as a renewable energy source. For example, the fragmentation of river systems due to the construction of dams hinders migratory species from reaching spawning areas [12,13]. Also, the increasingly market-oriented operation of hydropower plants leads to high flow variations in downstream river systems, a phenomenon called hydropeaking [14,15,16].

Another downside is total dissolved gas (TDG) supersaturation, which describes an excess of the sum of air-content gases within water above the usual concentration [17,18]. A recent study used a theoretical risk matrix to evaluate Norwegian hydropower plants with regard to their respective risk of introducing TDG supersaturated water into the downstream waterways. Their result shows that 28 % of all power plants are within high-risk classes, and a separate monitoring campaign confirmed that all of the power plants monitored introduced TDG saturation levels dangerous to the aquatic environment [19]. Nevertheless, long-known threshold levels of around 110 %TDG [20,21] are not enforced by Norwegian authorities, preventing a holistic overview of the problem of TDG supersaturation in Norway.

TDG supersaturation poses a danger to the aquatic environment due to gas bubble disease (GBD), which affects certain fish and aquatic invertebrates [22,23,24]. GBD is potentially lethal through gas embolism, but can introduce secondary effects due to a decreased immune system, hampered ability to dive, or scar tissue. Its extent is dependent on the type and life stage of the species, the TDG supersaturation level, the residence time, and the local water depth. Studies evaluating the response of certain fish species on avoiding TDG supersaturation by diving into deeper water regions have been inconclusive [21,25].

Most of the research on TDG supersaturation has been conducted in countries with a large share of electricity generation from hydropower, namely the USA, Canada, China, and Norway. Its cause was quickly identified as air intrusion into water, which can take place at different places in a hydropower plant [19,20]. When flowing over a large dam, turbulence leads to air being mixed into the water, which is then entrained into the downstream stilling basin, where it dissolves under the increased hydrostatic pressure. Similarly, air entering a hydropower plant’s waterways will be exposed to a pressure increase within the penstock. When exiting the stilling basin or passing the hydraulic turbine, the water is re-exposed to lower pressures, leaving it with an excess in dissolved gases, i.e., TDG supersaturated [26,27]. Methods to both prevent air intrusion or degas TDG supersaturated water downstream hydropower plants are well studied from a civil-engineering point of view [21,26,27]. Nevertheless, solutions need to be tailor-made for each power plant, and not all of them have the topological needs to retrofit the solutions offered. This is especially valid for Norwegian hydropower plants, which, due to topological conditions, differ a lot from high dams with big flow rates, as found in the USA, Canada, and China, in the sense of head size, volumetric flow, and number of secondary intakes [2,28]. A solution can be the investigation of technical methods to reduce the TDG saturation level downstream hydropower plants.

This study aims to shed light on three different methods known to increase the volumetric liquid–gas mass transfer coefficient, $k_{L}a$, namely acoustic degassing, aeration by gas bubbling, and liquid agitation. As liquid–gas mass transfer is dependent on multiple parameters, current investigations mostly focus on its enhancement for single gas species. In case of all gases in solution, i.e., TDG saturation, research is scarce, especially in case of TDG supersaturation. Therefore, the target is to utilize the change in TDG saturation level within the water to calculate $k_{L}a$. This is done by utilizing a unique experimental setup that is capable of generating a customized gas supersaturations in water, which subsequently is used to study the different degassing methods in a batch reactor. Each method will be described in detail in the subsections below.

The volumetric liquid–gas mass transfer coefficient consists of two parts, namely the partial transfer conductance $k_{L}$ [m*/*s] and the interfacial area a [m^2^*/*m^3^]. The former describes the speed of the mass transfer, while the latter shows how much area is available for dissolved gas species to move into or out of solution [29,30]. Each part can be targeted to enhance the overall liquid–gas mass transfer, but since the determination of the individual parts is difficult, the combined mass transfer coefficient will be used to compare the degassing methods’ effectiveness. Although the literature is rich in numerous articles concerning the study of material transfer in two-phase reactors (bubble columns, plate exchangers, etc.), much less information is available with regard to the gas–liquid equilibrium under ultrasound. If the importance of degassing during a sonochemistry operation is considered crucial, there are very few studies that deal with this subject in depth. A few works concern the effect of gas control [31], but almost nothing on exchanges and in particular on the volumetric mass transfer coefficient $k_{L}a$. Indeed, since the pioneering work of C. Petrier’s group [32] with one of the first mentions of degassing, only a few studies deal with the topic, and most of them in the very specific case of melted metals [33] or aluminum [34] and more generally in molten metals [35]. In aqueous solution, the influence of gas pressure has been investigated as a function of frequency and power [36] in order to detail their action into germination of cavitation bubbles [37]. It had also been shown in ionic liquids that a total quench of cavitation activity is possible while degassing deeply liquids with low volatile pressure [38]. Nevertheless, if the information on cavitation behavior are of primary interest, the driving forces between the dissolved gas and its partial pressure equilibrium are too weak to give clear insights into mass transfer mechanisms.

The very original contribution of the present work is to investigate the effect of ultrasound on mass transfer, compared to other techniques, with higher partial pressure gradients due to TDG supersaturation. With a custom-designed and built setup capable of producing gas supersaturation in water, $k_{L}a$ values obtained vary more significantly in the batch reactor used in this study, allowing us to appreciate the relationship between mass transfer and several parameters such as ultrasonic power or flow circulation. Each method will be described in detail in the subsections below.

### 1.1 Ultrasonic degassing

Applying an acoustic field within a liquid volume will superimpose a sinusoidal acoustic pressure wave on the ambient pressure [39,40]. If the field’s frequency is above 18 kHz to 20 kHz (the audible range of humans), it is referred to as ultrasound [41,42,43]. In the lower range of the ultrasonic frequency spectrum (20 kHz to 1 MHz), high powers in the kilowatt region can be applied, which is why it is referred to as power ultrasound [43,44,45]. This type is usually utilized in acoustic degassing processes.

Within the acoustic field in water, bubbles are formed due to spontaneous creation of singular bubbles at a nucleus being apparent in the liquid, continuous bubble appearance at objects submerged in the liquid or at walls, and fragmentation of bubbles already existing in the liquid [46]. After nucleation, the bubble will grow while still being within the acoustic field. The so-called Blake threshold describes whether a bubble will dissolve again after nucleation due to the liquid’s ambient pressure, or grow due to coalescence or rectified mass diffusion [47,48]. Rectified mass diffusion describes the process of uneven mass transfer between the gaseous and the liquid phase, which leads to bubble growth. This is triggered by area effects, which occur due to the oscillatory bubble motion, and shell effects, which describe the change in the thickness of the bubble shell [33,49]. Recent studies challenge bubble growth due to rectified mass diffusion due to non-linear theories for micro-bubbles opposing it. They introduce a rectified diffusion pressure as a threshold for bubble growth [50], which then follows the process of bubble nucleation growth due to coalescence or accumulation at the pressure antinodes, inertial bubble oscillation above the rectified diffusion threshold, and break up or multiplication at the fragmentation threshold [51,52].

Once at resonance size, a bubble can either become unstable and collapse, or stable oscillating [39,53,54]. The latter is the primary driver of degassing using ultrasound, as it, once overcoming buoyancy, rises and leaves the liquid at its surface. The number of these so-called degas bubbles [55] is directly related to the power within the acoustic field [56,57,58]. The mass transfer coefficient during ultrasonic degassing decreases when the sonicated volume is increased [58]. Similarly, the existence of large bubbles influences the mass transfer negatively due to dampening effects, as they hamper the acoustic wave propagation [59]. Moreover, the cavitation intensity and therefore the degassing efficiency decreases at high TDG saturation levels [60]. Nevertheless, applications for ultrasonic degassing are found within various industries, such as in metallurgy (e.g., liquid metal degassing), chemical industry (e.g., degassing resin solutions or transformer oil), electrochemistry (e.g., deposition of coatings), or food processing (e.g., degassing of food oils or beverages) [33,61]. All these use the advantages of ultrasonic degassing, which are fast and thorough degassing, environmental friendliness, efficiency, and economic viability [62,63].

### 1.2 Aeration

Introducing gas bubbles into a liquid can lead to an alteration of its dissolved gas content. Especially when treating gas supersaturated water, the difference between the dissolved gas pressure and the bubble’s internal pressure drives gas species out of the solution. Many studies focus on degassing of a static volume of TDG supersaturated water. An aeration water column was used by Ou et al. (2016) to study the dissipation rate of TDG supersaturation in dependence of gas flow rate, insertion depth, and diffuser pore size. They revealed a correlation between increased degassing and higher gas flow rates, lower water depth, and smaller diffuser pore size [64]. Furthermore, the gas retention time was higher when increasing the insertion depth, and bubble sizes were smaller with lower gas flow rates, both leading to an increase in mass transfer. In a later study, the influence of the gas diffuser’s size was found to positively influence the degassing rate [65]. Yao et al. (2023) conducted similar experiments and developed a mathematical model that enables numerical simulation of the aeration process [66]. A reason for the results of the aforementioned studies is the increase in the gas–liquid interfacial area when flushing gas bubbles into the water body. This works theoretically as well, as shown by Zhang et al. (2017). They described aeration by gas bubbling as potentially very effective in degassing TDG supersaturated water. Moreover, they determine the optimal bubble diameter for degassing purposes to be about 0*.*1  mm [67].

Few studies are known to attempt degassing by aeration on flowing water. Lichtwardt and Murphy (2001) conducted both laboratory experiments and field tests to determine the effectiveness of gas bubbling to mitigate TDG supersaturation in water downstream a water treatment plant. Following their results, the main parameter affecting the degassing is the gas bubble size, which in turn depends on the diffuser’s pore size, the surface tension at the gas–liquid interface, and the gas density. They conducted additional experiments flushing oxygen instead of air, which led to an increased retention time and surface area, thus positively influencing the degassing rate. Another positive effect was observed in the increased nitrogen removal from the water body, with a total reduction of up to 34 %. Pure air bubbling had a negligible effect during field tests [68].

### 1.3 Liquid agitation

Is the dissolved gas content in water higher than supposed at atmospheric pressure, i.e., supersaturated, a natural drive to equilibrium will occur. Without the introduction of gas bubbles, the only interface for dissolved gas species to leave the water body is the water surface. If the water body is static and not agitated, a saturation gradient develops over time due to limited movement potential of the dissolved gas species. Then, the upper part of the water body, close to the water surface, will be at equilibrium, while the gas saturation will increase with the water depth.

Introducing turbulence in the liquid increases mixing processes, which in turn replenishes the gas saturation at the water surface. In addition, turbulence will increase the water surface due to wave formation, thus providing a larger liquid–gas interfacial area. Therefore, the liquid–gas mass transfer is enhanced [69]. Few studies shed light on utilizing this mechanism to mitigate TDG supersaturation. Shen et al. (2014) investigated the influence of temperature and turbulence on the dissipation of TDG supersaturation. Their findings show a direct correlation of the dissipation rate with increased temperature and turbulence [70]. A similar effect of the turbulence intensity was presented by Feng et al. (2014) [71]. This is also proven for natural rivers, where increased river turbulence affects the dissipation of the TDG saturation positively [72]. While the riverbed characteristics will lead to the generation of turbulence within the water body, external factors like wind can increase turbulence close to the water surface as well. The enhancement of the mass transfer and therefore the degassing process in this case is similar to turbulence being introduced internally or by stirring [73].

## 2 Methods and experimental details

To study the enhancement of liquid–gas mass transfer in TDG supersaturated water, a unique experimental setup was designed and built at the Norwegian University of Science and Technology (NTNU). This setup consists of a pressure vessel used to increase the TDG saturation in water, and a batch reactor in which the actual degassing experiment is conducted. To compare the mass transfer coefficient, the TDG saturation is monitored throughout the degassing phase of the experiment. The equipment used and the method to determine the liquid–gas mass transfer coefficient are described below. All experiments were conducted in the Hydrogen Energy and Sonochemistry Laboratory at NTNU.

### 2.1 Measurement equipment

The TDG saturation level in the batch reactor is monitored using a Weiss saturometer. This sensor is based on a semipermeable membrane which allows for the diffusion of dissolved gases and water vapor [74]. If the dissolved gas pressure within the liquid rises, osmosis will lead to a simultaneous increase of the pressure within the sensor’s silastic tubing, which is detected by a built-in pressure sensor. The reading is a direct measurement of the total gas pressure, $p_{TGP}$. During all experiments, a PT4 TGP sensor supplied by InWater Technologies was used, which was calibrated using the pressure chamber method described by Pleizier et al. (2021) [75]. Its measurement accuracy is given as ±2%TDG, and the results show a standard deviation of ±0.5%TDG, leading to a total measurement error of ±2.07%TDG. A GE UNIK 5000 pressure sensor (PTX 5072) with a range of 0 bar absolute to 3 bar absolute was utilized to determine the atmospheric pressure, $p_{atm}$. Both readings resulted in the TDG saturation level, as shown in Eq. (1).

(1) $$\begin{array}{c} S_{TDG}=\frac{p_{TGP}}{p_{atm}}\cdot 100 \end{array}$$

At equilibrium, a reading results in 100 %TDG. One experimental challenge of TDG sensors is a long response time in the range of 5  min to 30  min [75,76]. In addition, the $p_{TGP}$ gives no information about the share of different gas species in solution, e.g., N_2_ or O_2_, which is why further equipment is necessary for an in-depth study. Nevertheless, this method represents the only possibility to measure the TDG saturation level real-time and in situ, and gives the possibility to determine the liquid–gas mass transfer. Throughout all experiments, the temperature in the liquid batch was monitored using a PT100 resistance thermometer. All data is recorded using NI LabVIEW and NI DAQ systems.

### 2.2 Pressure vessel

**Fig. 1:**
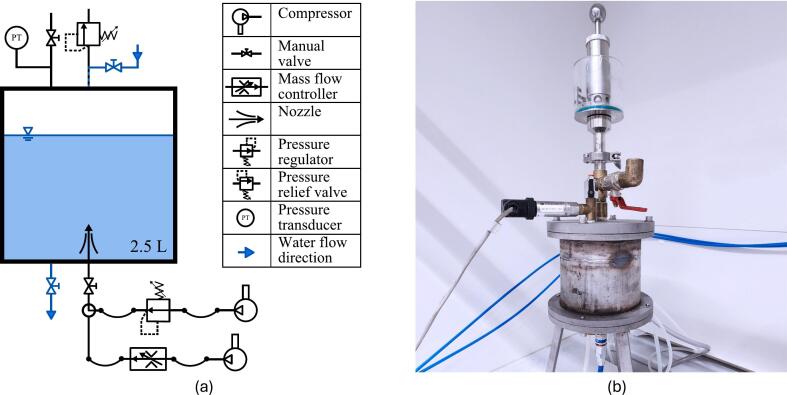
Schematic (a) and picture (b) of the pressure vessel used in NTNUs Hydrogen Energy and Sonochemistry Laboratory.

The transfer of gas into a liquid is highly dependent on the ambient pressure. A pressure vessel was designed with the intent to prepare a volume of $V_{pv}$ *≈* 2*.*5 L TDG supersaturated water for the mass transfer enhancement experiments. It consists of a cylindrical steel container with an air inlet at the bottom and a Brewtools pressure relief valve on top. The vessel is filled with pure water from the top, the vessel is closed, and gas is flushed into the system through a porous media (pore size: 0.5 µm) at the bottom. This introduces a large amount of small bubbles into the liquid. In addition, the ambient pressure will rise until the relief valve opens at a threshold of 2 bar. Then, a constant flow of gas through the liquid body enables dissolution, until a new equilibrium is reached. The ambient pressure inside the vessel is monitored using a GE UNIK 5000 pressure sensor (PTX 5072). A valve at the bottom allows emptying the vessel. A schematic and a picture of the pressure vessel are displayed in Fig. 1.

The gas pressure into the system is set to a value higher than the relief pressure of the valve to ensure sufficient gas flow through the water. It is regulated to 1*.*5 bar using a Festo MS6-LRP pressure regulator (0*.*05 bar to 2*.*5 bar), which is fed by an in-house compressed air system. The ambient pressure is recorded with a sampling frequency of 1  Hz. After waiting for 2  h, the gas supply is stopped, and the ambient pressure is slowly decreased to atmospheric pressure through a valve on top of the pressure vessel. The TDG supersaturated water flows through the bottom valve and a 1  m long hose into a 1 L graduated cylinder. Using the rather long hose allows for smooth transfer of the water by decreasing pressure losses occurring at the outlet, which otherwise could lead to natural degassing. A measurement of the TDG saturation level within the pressure vessel could not be conducted due to the pressure-tightness of the vessel which prevents direct access to the liquid bulk.

### 2.3 Equipment for mass transfer enhancement

All experiments on the enhancement of liquid–gas mass transfer are conducted within a Meinhardt Ultrasonics 1 L batch reactor. It is made of double-walled glass to allow for cooling of the liquid batch. Inlets on the top and the bottom of one side are used to circulate cooling water, while two inlets on the other side give direct access to the liquid batch. Top and bottom of the reactor are open as well. The bottom is used to attach ultrasonic transducers, while the top allows for filling and inserting sensors. The tests of aeration and liquid agitation, as well as the combined method tests, were repeated once. The measurements to determine the effect of the acoustic frequency were conducted twice, while the effect of the ultrasonic power was tested multiple times at higher powers.

**Fig. 2:**
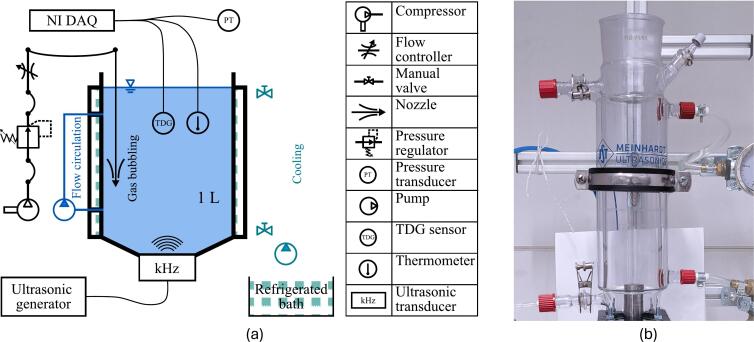
Schematic (a) of the experimental setup and a picture (b) of the batch reactor at NTNUs Hydrogen Energy and Sonochemistry Laboratory.

Cooling water is stored in a Neslab RTE-221 refrigerated bath and circulated by a Marco Fluidtech UP3/E electronic gear pump. The cooling temperature is dependent on both the ultrasonic transducer and its respective power and is determined empirically. During the experiments, the sampling frequency was increased to 5 Hz. Fig. 2 shows a schematic overview of the batch reactor, including all the experimental equipment used during the individual experiments.

#### 2.3.1 Ultrasound

Three commercially available ultrasonic transducers are used to sonicate the liquid in the batch reactor. Two of them are manufactured by Meinhardt Ultrasonics, while the third one is made by SinapTec. By using those three transducers, the influence of the frequency on the degassing efficiency is determined. The tested frequencies are 20 kHz, 40 kHz, and 580 kHz. In case of the last frequency, the transducer is actually a multi-frequency transducer, but only the lowest one available is tested. All transducers are operated using different generators and can achieve different maximum acoustic powers. In addition, they differ by type, which means that they feature different sizes of their transmitting surface, which in turn results in different mountings for the transducers. A summary of the properties of the transducers is given in Table 1.

**Table 1:** Properties of the three ultrasonic transducers used during the batch degassing experiments.

Manufacturer	Transducer type	Frequency tested [kHz]	Max. acoustic amplitude [W]
SinapTec	horn	20	400
Meinhardt Ultrasonics	flat	40	50
Meinhardt Ultrasonics	flat	580	250

**Fig. 3:**
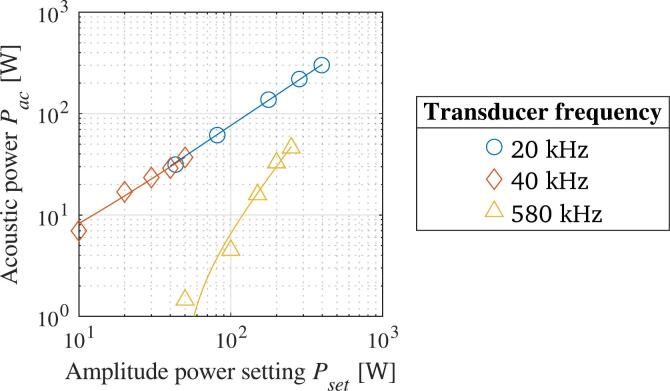
Acoustic power measurements over amplitude power setting for the different ultrasonic transducers.

Due to the large differences in power, the transducers are tested prior to the degassing tests with regard to their transmitted acoustic power. This is done using the calorimetric method, which uses the heat being dissipated by the transducer into the water as a proxy for the acoustic power, assuming that all the acoustic energy absorbed by the water is transformed into heat [77,78]. 1 L of water is sonicated for 5  min without cooling, and each generator is tested at 5 different acoustic amplitudes. The temperature slope measured using the thermometer yields the acoustic power for the respective amplitude setting. Fig. 3 shows the resulting, averaged data points for the three transducers and the respective trendlines.

The figure reveals that only a limited area is available where the transmitted powers of the transducers overlap. In this area, the influence of the frequency on the degassing of TDG supersaturated water is tested. The transmitted acoustic power is set to 37  W for all transducers. The batch is sonicated for 10 min at a constant temperature of 20 °C.

Another experiment aimed at studying the influence of the transmitted acoustic power on the mass transfer enhancement. As the SinapTec 20 kHz transducer had the highest range of amplitude, it was chosen for this study. The transducer amplitude was varied between 50 % and 100 % to measure its effect. The other experimental parameters were as before.

#### 2.3.2 Agitation

The batch was agitated by circulating the liquid volume within the reactor using external pumps. To achieve a wide range of circulation speeds, two pumps were used: a Marco Fluidtech UP2/E electronic gear pump with a maximum flow rate of 10 L*/*min, and a Shenchen M6-6L peristaltic pump with a maximum flow rate of 6 L*/*min. Due to head losses stemming from the pump placements, the pumps were calibrated prior to the experiments.

The flow in the reactor was circulated either concurrent or countercurrent to the degassing movement, i.e., from the bottom to the top or vice-versa. The electronic gear pump was operated with flow rates of 1*.*92 L*/*min, 3*.*82 L*/*min, and 5*.*30 L*/*min in the countercurrent and the same in addition to 6*.*20 L*/*min for the concurrent flow. The peristaltic pump circulated water at flow rates of 0*.*5 L*/*min, 1*.*0 L*/*min, and 1*.*5 L*/*min in the countercurrent flow, but only at 1*.*5 L*/*min in the concurrent flow. To compensate for the added volume of the pump and hoses, 0*.*8 L of TDG supersaturated, pure water was used during the experiments.

Moreover, ultrasonication and flow circulation were combined to see whether an additional enhancement of the liquid–gas mass transfer was achieved. For this, the SinapTec 20 kHz transducer was used at 100 % amplitude in combination with the two pumps mentioned above. The tested flow rates were 1*.*5 L*/*min, 1*.*92 L*/*min, 3*.*82 L*/*min and 6*.*20 L*/*min in the countercurrent and 1*.*5 L*/*min, 1*.*92 L*/*min, 3*.*82 L*/*min and 5*.*30 L*/*min in the concurrent flow circulation. The timing for all experiments was similar with 10  min of degassing at 20 °C.

#### 2.3.3 Aeration

The aeration by gas bubbling was achieved by utilizing a gas diffuser and a flow regulator. The effect of the bubble size was investigated by using diffusers with a different pore size: a sealed, synthetic tube with a pore size of 2  mm, an ace gas diffuser with a pore size range of 145  µm to 174  µm, and an oxygenation stone with a pore size of 0.5  µm. Air is used for bubbling, which is provided by an in-house compression system, regulated down to 0*.*5 bar using a Festo MS6-RLP, and then further regulated to a flow rate between 1 L*/*min to 10 L*/*min using a Key Instruments FR2000 variable area flow meter. During the experiments, the respective diffuser was inserted from the top of the reactor and placed close to the bottom. The gas flow rate was varied between 1 L*/*min and 10 L*/*min for the synthetic tube and the oxygenation stone, while only 1 L*/*min and 4 L*/*min were used for the ace gas diffuser. This was due to the limited surface area of the latter diffuser.

The aeration using the oxygenation stone was combined with ultrasound using the SinapTec 20 kHz transducer as well. Here, the transducer was operated at 100 % amplitude, while the gas flow rate was set to 1 L*/*min and 10  L/min, respectively. Again, all experiments were conducted for 10  min of degassing time, at 20 °C.

### 2.4 $k_{L}a$determination

To allow for comparison between all the methods tested, the liquid–gas mass transfer coefficient, $k_{L}a$, was calculated from the change in TDG saturation within the batch reactor. This in turn was calculated following Eq. (1), using the measurements of both the TGP and pressure sensor, as described in Section 2.1. The mass transfer situation within the batch reactor is highly dynamic, which prevents the calculation of $k_{L}a$ by simply taking the difference of initial and final TDG saturation. Instead, the coefficient is derived from the measurement data following the mass balance in the case of a static liquid.

(2) $$\begin{array}{c} -k_{L}adt=\frac{dS_{TDG}\left(t\right)}{S_{TDG}\left(t\right)-S_{TDG}^{*}} \end{array}$$

Here, the subtraction of the baseline TDG saturation is included into the derivation term.

(3) $$\begin{array}{c} -k_{L}adt=\frac{d\left(S_{TDG}\left(t\right)-S_{TDG}^{*}\right)}{S_{TDG}\left(t\right)-S_{TDG}^{*}} \end{array}$$

This equation has known boundaries at *t* = 0, where the sensor reading is stable at the highest TDG saturation, $S_{TDG,max}$, and the actual measured TDG saturation value at a time t>0. Now, both sides of Eq. (3) can be integrated with regard to time and saturation.

(4) $$\begin{array}{c} -k_{L}at=\int_{S_{TDG,max}}^{S_{TDG}\left(t\right)}\frac{d\left(S_{TDG}\left(t\right)-S_{TDG}^{*}\right)}{S_{TDG}\left(t\right)-S_{TDG}^{*}}\\ =\ln\left(\frac{S_{TDG}\left(t\right)-S_{TDG}^{*}}{S_{TDG,max}\left(t\right)-S_{TDG}^{*}}\right) \end{array}$$

Eq. (4) shows that the liquid–gas mass transfer coefficient is the negative slope of the logarithmic difference on the right-hand side, and is only dependent on the TDG saturation at time t.

As described in Section 2.1, the TGP sensor’s response time is slow. The change in the TDG saturation within the reactor is faster than this response time, so a time constant, $\delta_{TDG}$, is determined to compensate for this. The sensor’s response to the change in the TDG saturation level is used to calculate it. The response of the sensor, i.e., the difference between the stable values before and after the change in TDG saturation, $\Delta S_{\delta }$, is used to determine the time constant, which is obtained using the following equation [79].

(5) $$\begin{array}{c} {\left(1-\exp\left(-\frac{t}{\delta_{TDG}}\right)\right)}_{t=\delta }\Delta S_{\delta }=63.21\%\cdot \Delta S_{\delta } \end{array}$$

From four steps with increasing and four with decreasing TDG saturation, the averaged time constant is calculated to be $\delta_{TDG}=63.8s$. This value is used to correct the measured TDG saturation value using the following equation.

(6) $$\begin{array}{c} S_{TDG}^{\prime }=S_{TDG,0}+\Delta S_{TDG}\left(1-\exp\left(-\frac{t}{\delta_{TDG}}\right)\right) \end{array}$$

Here, $S_{TDG}^{\prime }$ is the corrected, $S_{TDG,0}$ is the initial, and $\Delta S_{TDG}$ is the difference between the initial and the measured TDG saturation at time *t*. Afterwards, the TDG saturation curve is smoothened to remove outliers using the movmean-function in MATLAB with a sliding window of 40 elements. The resulting data is used in Eq. (4). Finally, the polyfit-function in MATLAB is used to determine the linear slope of the real part of the resulting logarithmic difference values. This slope represents the negative liquid–gas mass transfer coefficient, $k_{L}a$, which is used to compare the mass transfer enhancement.

## 3 Results and discussion

Following the experimental steps presented in the previous section, the liquid–gas mass transfer coefficient is determined. All the results are presented and discussed within this section for each of the respective methods. Even though some of the experiments were conducted using multiple measurements at the same operational points, the resulting graphs presented in this section display individual measurements only.

### 3.1 Ultrasound

**Fig. 4:**
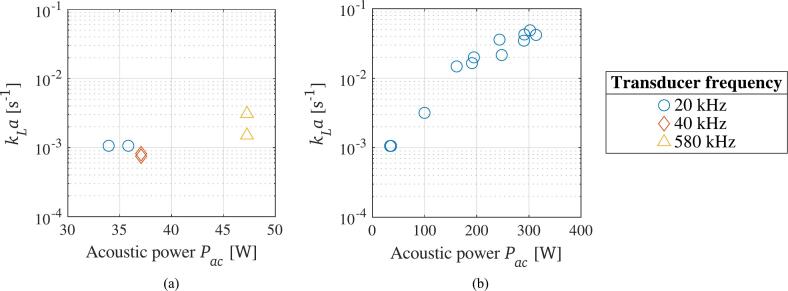
Influence of frequency (a) and acoustic power (b) on the volumetric liquid–gas mass transfer coefficient during ultrasonic degassing.

The effect of the frequency and the acoustic power of the three ultrasonic transducers on the liquid–gas mass transfer were investigated. The resulting $k_{L}a$ values for the different frequencies are displayed in Fig. 4a. It is apparent that all three frequencies lead to a similar mass transfer, even though the 580 kHz transducer achieves slightly higher values, with a mean $k_{L}a$ of 2*.*29 *×* 10^−3^ s^−1^, while the 20 kHz and 40 kHz transducer achieve a mean $k_{L}a$ of 1*.*06 *×* 10^−3^ s^−1^ and 0*.*78 *×* 10^−3^ s^−1^, respectively. The 580 kHz transducer is also the one using a somewhat higher acoustic power. Still, the results are comparable to findings presented by Asakura et al. (2022), who found little to no difference in the degassing rate of a 22 kHz and a 43 kHz transducer, while frequencies above 200 kHz were found to degas water faster [36]. Somewhat problematic is the use of different types of transducers powered by different generators. As the surface area of the 20 kHz transducer is much smaller than the ones of the 40 kHz and 580 kHz transducers, much more power per surface area is released, leading to a higher acoustic streaming effect within the liquid. This can lead to a slight increase in the mass transfer for the first-mentioned transducer. Yet, the acoustic streaming is more visibly apparent towards higher acoustic powers.

The effect of the acoustic power on the degassing efficiency is visible in Fig. 4b. A clear trend of higher mass transfer towards higher acoustic powers is indicated, with the mean $k_{L}a$ of the four measurements at the highest amplitude being 42*.*13 *×* 10^−3^ s^−1^. Similar results have again been reported by Asakura et al. (2022), but the resulting mass transfer in the present study is one order of magnitude higher than their findings [36]. The reason for the increased mass transfer is found in the increased cavitation intensity, which in turn promotes the emergence of degassing bubbles [80].

### 3.2 Liquid agitation

**Fig. 5:**
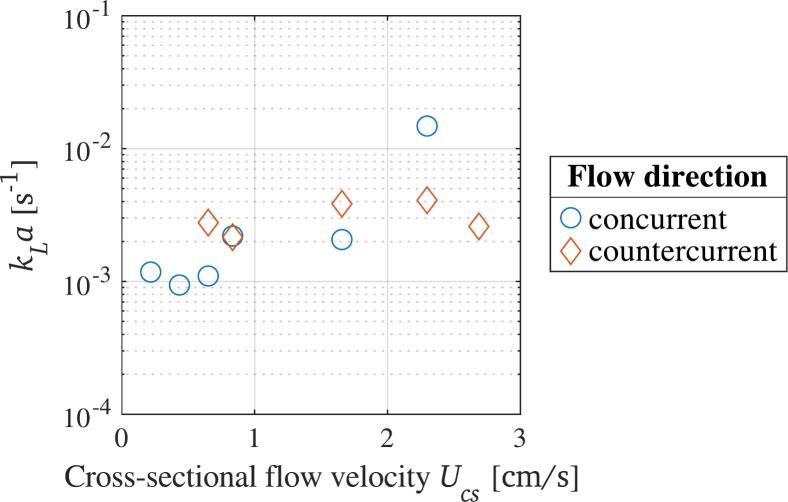
Volumetric liquid–gas mass transfer coefficient in dependence of the cross-sectional flow velocity during flow circulation in the reactor.

The enhancement of the liquid–gas mass transfer by liquid agitation was investigated by circulating water within the batch reactor. The resulting $k_{L}a$ values are displayed in Fig. 5 over the cross-sectional flow velocity, $U_{\mathrm{cs}}$. The velocity is calculated by dividing the circulation pump flow rate $Q_{p}$ by the cross-sectional area of the batch reactor. In the concurrent flow situation, higher flow velocities in the reactor lead to higher mass transfer. An outlier at the highest flow velocity in this case is not shown, as it leads to an-naturally high values. It is explained by the pressure drop within the circulation pump during concurrent flow circulation, which enhances the natural degassing. For the countercurrent flow circulation, the trend is rather constant throughout the tested flow velocities. The mean $k_{L}a$ value in this case is 3*.*09 *×* 10^−3^ s^−1^, whereas the mean $k_{L}a$ in case of concurrent flow is at 1*.*50 *×* 10^−3^ s^−1^ (excluding the outlier). Therefore, both methods lead to mass transfer in the same order of magnitude, with slightly higher values in case of the concurrent flow circulation. The slight difference between the two cases is explained by the increase in residence time for gas bubbles forming at nuclei within the batch reactor, which are entrained longer in case of countercurrent flow circulation and therefore increase the interfacial area longer than in case of concurrent flow. A recent study by Pincovschi et al. (2025), where oxygen mass transfer in a stirred tank was studied, shows $k_{L}a$ values in a similar range of magnitude as achieved in the present study [81].

### 3.3 Aeration

**Fig. 6:**
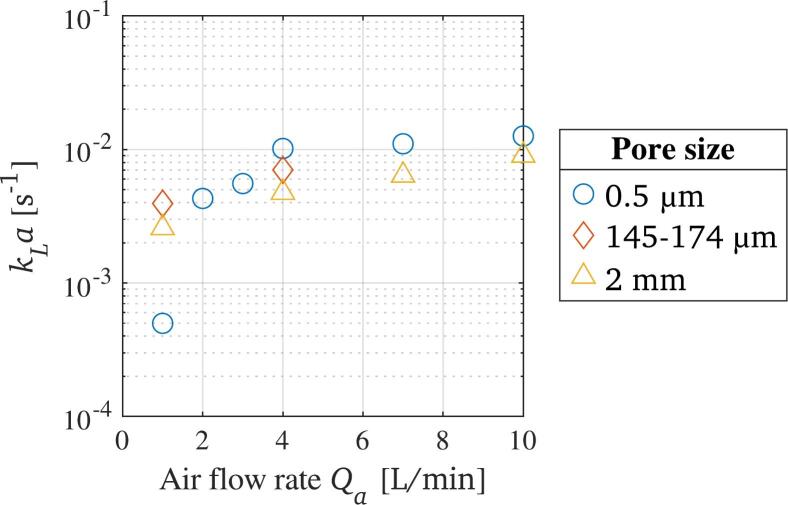
Volumetric liquid–gas mass transfer coefficient during aeration in the reactor, in dependence of gas diffuser pore size and air flow rate.

Aeration as a method to enhance liquid–gas mass transfer was tested by bubbling air into the reactor. The resulting $k_{L}a$ values over the air flow rate, $Q_{a}$, are displayed in Fig. 6. The pore sizes in the figure’s legend correspond to the oxygenation stone, ace gas diffuser, and synthetic tube, respectively, that were used for the gas bubbling. A trend of high mass transfer towards high air flow rates is apparent for all the diffusers. For the synthetic tube, this trend is almost linear, while the mass transfer coefficient for the oxygenation stone rises sharply at lower air flow rates and moves towards a plateau at higher air flow rates. In addition, a reduction of the pore size leads generally to an increase in mass transfer. The outlier for the lowest air flow rate tested with the oxygenation stone shows a $k_{L}a$ value of one order of magnitude lower than the other results. This can be explained by the visually observed emergence of very few, large bubbles at these experimental settings, presumably occurring due to preferred gas channels within this gas diffuser, resulting in a decreased mass transfer due to a lower interfacial area.

Similar increases in the mass transfer in dependence of bubble size and number are found in literature. As the interfacial area increases when decreasing the bubble size at a simultaneous increase in the bubble number, the liquid–gas mass transfer coefficient will be higher [82,83]. Furthermore, an increasing gas flow rate will induce agitation to the liquid, leading to mixing processes and turbulence, which in turn enhance the mass transfer further.

Reports from literature show benefits of applying micro or nano bubbles to enhance liquid–gas mass transfer. These feature gas bubbles that are sized 10 to 50 µm or <200 nm, respectively. At these sizes, the bubbles’ rising velocity is drastically reduced, leading to large retention times. In addition, their large surface to volume size is beneficial to the liquid–gas mass transfer. Liquid-gas mass transfer coefficients reported are in the order of magnitude, but larger than the results of the aeration tests, with peak values reaching 19.26 × 10^-3^ s^−1^ [84] or 64.50 × 10^-3^ s^−1^ [85]. Yet, the intended degassing application of the aeration within the present project does not allow the use of bubbles below a certain threshold diameter, due to hydrostatic pressures that will dissolve smaller bubbles.

### 3.4 Combined methods

**Fig. 7:**
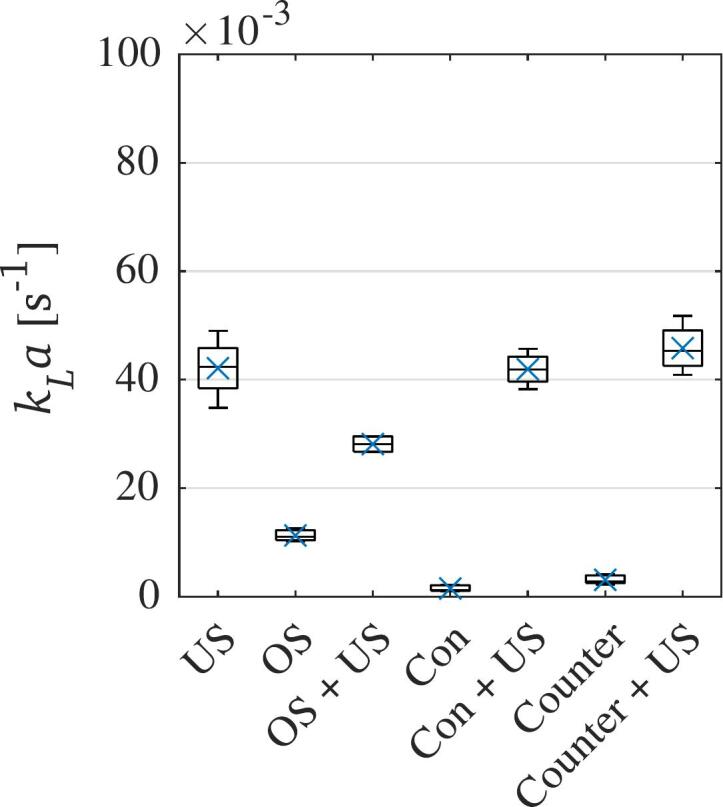
$k_{L}a$ values obtained for single methods and combination with 20 kHz ultrasound at 100 % amplitude.”US” − sonication,”OS” − aeration with oxygenation stone,”Con” &”Counter” − liquid agitation through con- & countercurrent flow circulation.

At the highest $k_{L}a$ values achieved for liquid agitation and aeration, a combination with the highest-power sonication using the 20 kHz transducer is tested to study possible promotive effects. In case of aeration, the oxygenation stone at gas flow rates of 1 L*/*min and 10 L*/*min is used, while for flow circulation, several flow rates in both con- and countercurrent flow situations are tested. As the resulting trends show results being achieved at similar levels, the results are averaged and plotted together to allow for easier comparability. In Fig. 7, the highest achieved $k_{L}a$ values for each individual method is plotted as well.

It is apparent that the high-power ultrasound achieves much higher liquid–gas mass transfer coefficient values than the best methods for aeration and liquid agitation. Combining the latter methods with ultrasound increases the mass transfer when compared to the methods alone. When compared to the $k_{L}a$ values achieved with ultrasound alone, a combination with aeration achieves a lower mass transfer. This can be explained by the disturbance of the acoustic field by the presence of large air bubbles, which dampen the acoustic waves. Combining ultrasound with liquid agitation gives similar results as ultrasound alone, with even slightly higher mass transfer being achieved in the countercurrent flow circulation scenario. In this case, the velocity field is highly disturbed by both acoustic streaming and the flow circulation, while the acoustic waves are not deflected [86,87]. Therefore, in the countercurrent flow scenario, the degassing bubbles developing within the acoustic field are entrained longer within the liquid batch and the acoustic field. This leads to a longer residence time and therefore larger degassing, similar to the results from liquid agitation only.

### 3.5 Discussion

As mentioned in the previous section, the highest values for the liquid–gas mass transfer coefficient were observed during ultrasonication at 20 kHz and high acoustic powers. This implicates a strong influence of the acoustic degassing on both the partial transfer conductance, $k_{L}$, and the interfacial area, a, which distinguishes this method from the other two which mainly influence *a*. It cannot be ruled out that higher frequencies at the same power might enhance the mass transfer even more, but restrictions in the experimental equipment prevented further verification of this assumption. In addition, increasing the frequency can potentially result in a higher number of unstable cavitation events, which would lead to unwanted chemical reactions. The design of the three transducers used in the experiments also has a potential effect on the results, as differences in the emitting surface area led to variations in the transmitted power density. Furthermore, due to their respective operating frequency, a difference in transducer thickness may have impacted the thermal stability and operational efficiency. Nevertheless, a comparison with the findings of Asakura et al. (2022) shows good agreement with the general trends observed in this study [36].

Liquid agitation through flow circulation in the batch reactor increased the mass transfer coefficient as well, but the $k_{L}a$ values achieved were one order of magnitude smaller than the ones achieved with high-power ultrasound. The direction of the flow showed that countercurrent flow was more beneficial for the mass transfer than concurrent flow, as it increased the residence time of gas bubbles within the liquid. Experimental constraints, such as natural degassing during certain operational points of the pumps, need to be addressed in future setups to improve the accuracy of the experimental outcomes at high circulation flow rates. Aeration by gas bubbling in the liquid enhanced the mass transfer as well, with a linear increase at lower and a plateau effect at higher gas flow rates. The pore size of the gas diffuser proved to be influential, as smaller pores generate finer bubbles, by such increasing the interfacial area and improving the mass transfer. However, even the highest mass transfer coefficient values achieved by aeration were only one-quarter of those obtained with high-power ultrasonication.

The combination of ultrasound and gas bubbling resulted in a drastic decrease in mass transfer compared to ultrasound alone. Several effects lead to this decrease, such as the disruption of cavitation-induced degassing bubbles, as the larger bubbles introduced by aeration cause coalescence and removal of bubble nuclei from the liquid. Furthermore, the acoustic field within the batch reactor was relatively small compared to the volume of bubbles introduced by the gas diffuser, so a larger reactor volume and a broader acoustic field at the same power level may lead to different results. Nevertheless, the $k_{L}a$ values of the present study align with values reported by Sajjadi et al. (2017), who studied acoustic stirring in an aerated reactor [88].

By contrast, combining ultrasound and liquid agitation by countercurrent flow circulation led to a slight increase in the mass transfer coefficient. This improvement was due to the increased residence time of bubbles in both the liquid and the acoustic field. This enhancement is not present in the case of concurrent flow circulation and sonication of the liquid batch.

The practicability of a final degassing application has to be evaluated with regard to energy requirements, as this is an important economic factor and can influence the decision-making of the end user. While the power consumption of the utilized devices was not measured during the experiments, the maximum power requirements give an indication and enable comparison. The maximum power per volume ratio during ultrasonication is 1000  W/L, while the two pumps used during liquid agitation have 75  W/L and 225  W/L in case of the electronic gear and peristaltic pump, respectively. The power required by the in-house pressure system could not be acquired and is not representative. Therefore, the power of small air compressors was used as an estimation, leading to a power per liquid of approximately 250  W/L. It is obvious that ultrasonication requires much more power than the other methods. Yet, when adjusted for the power to $k_{L}a$ ratio, ultrasonic degassing stands out as most efficient. The ratios for the best $k_{L}a$ values reached are 17.81 kWs, 19.35 kWs, and 22.12 kWs for ultrasonication, liquid agitation, and gas bubbling, respectively.

An important limitation of the experimental setup was the size of the TGP sensor in comparison to the reactor volume. Since measurements of the TDG saturation level had to be conducted in situ, the sensor reduced the liquid volume by approximately 25 %, therefore effectively reducing the active reaction zone. Moreover, the sensor body partially obstructed flow circulation, ultrasonic streaming, and bubble movement, which potentially affected the experimental outcomes. The long sensor response time posed an additional challenge, which was overcome by manually adjusting the measurement values using the sensor time constant. However, since all tested methods were affected in a similar manner, the comparability of results remains given.

**Fig. 8:**
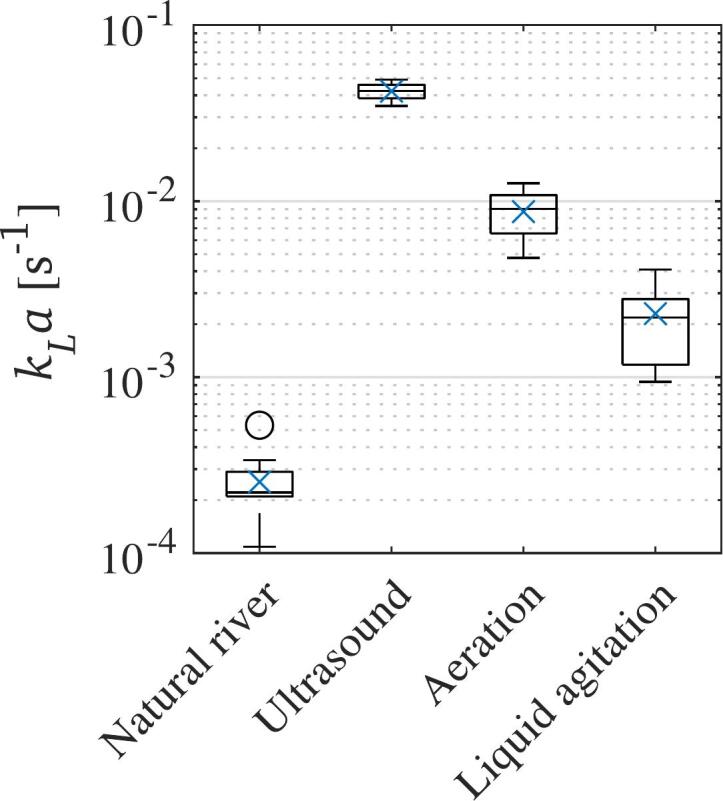
Comparison of $k_{L}a$ values for natural degassing in a river compared to the evaluated methods.

Finally, the results obtained in this study are compared to the $k_{L}a$ during the natural degassing process of TDG supersaturated water in a river downstream a Norwegian hydropower plant. Those results were obtained from measurement data provided by The Norwegian Research Center (NORCE) [89]. A direct comparison is visible in Fig. 8. It is apparent that all methods tested enhance the liquid–gas mass transfer by one to two orders of magnitude when compared to the natural degassing process. This comparison does not take into account the effect of the static liquid in the batch reactor, nor the initial TDG saturation level. Nevertheless, the results seem promising in finding a technical solution that can help mitigate TDG supersaturation downstream hydropower plants.

## 4 Conclusion

All tested methods enhanced the liquid–gas mass transfer coefficient in a small batch reactor when compared to natural degassing. Ultrasonic degassing was the most efficient method to enhance the mass transfer, while aeration by gas bubbling and liquid agitation through flow circulation led to a smaller improvement. It is important to note that, starting from TDG supersaturation, it is possible to quantify the global decrease of dissolved gas content up to an equilibrium, showing a real dependence on the acoustic power.

The combination of aerating and agitation techniques with ultrasound produced mixed results, as gas bubbling hindered the ultrasonic degassing effect, while con- and countercurrent flow provided similar results as ultrasound alone, with the latter showing a small but measurable enhancement.

These findings have several practical implications. The acoustic degassing process could be further optimized for applications such as water treatment, hydropower environmental mitigation, and industrial gas–liquid reactions. However, more investigation of the acoustic parameters is needed to avoid unwanted cavitation-related side effects, such as excessive heating or chemical reactions. In addition, scale-up challenges remain, particularly with regard to reactor design, introduction of continuous liquid flow, and sensor placement.

Future research should focus on both the effect of the acoustic frequency above 20 kHz at high acoustic powers, as well as an energy efficiency assessment is needed to evaluate the feasibility of the studied methods in large-scale operations. Moreover, the influence of the acoustic frequency on enhancing the degassing efficiency for different air-content gases could offer the possibility of targeting a preferred gas species. Finally, finding a reactor design that allows for advanced placement and combination strategies of both liquid agitation and aeration with ultrasound can enhance the overall enhancement of liquid–gas mass transfer and lead to an advancement of technologies across multiple industrial and environmental applications.

In the scope of the DeGas project, scaling is of utmost importance. This includes both an increased volume of TDG supersaturated water and a move towards continuous degassing of water flowing past a degassing device. The installation of an experimental test rig at the Waterpower Laboratory at NTNU will give further insights and will be closer to the final application within a hydropower plant.

## CRediT authorship contribution statement

**W. Ludwig Kuhn:** Writing – review & editing, Writing – original draft, Visualization, Software, Methodology, Investigation, Formal analysis, Data curation, Conceptualization. **Jean-Yves Hihn:** Writing – review & editing, Supervision, Data curation, Conceptualization. **Bjørn Winther Solemslie:** Writing – review & editing, Supervision. **Ole Gunnar Dahlhaug:** Writing – review & editing, Supervision, Resources, Project administration, Funding acquisition.

## Declaration of competing interest

The authors declare that they have no known competing financial interests or personal relationships that could have appeared to influence the work reported in this paper.

## References

[1] IPCC, Climate Change 2013: The Physical Science Basis. Contribution of Working Group I to the Fifth Assessment Report of the Intergovernmental Panel on Climate Change, Cambridge University Press, Cambridge, United Kingdom and New York, NY, USA, 2013.

[2] Graabak I., Jaehnert S., Korpås M., Mo B. (2017). Norway as a battery for the future European power system—impacts on the hydropower system. Energies.

[3] WBG, World Bank Climate Change Knowledge Portal, The World Bank Group. Accessed: May 04, 2023. [Online]. Available: https://climateknowledgeportal.worldbank.org/.

[4] NVE, Overview of Norway’s Electricity History, Norges vassdrags- og energidirektorat, Oslo, Norway, 15, 2017. Accessed: Oct. 20, 2023. [Online]. Available: https://publikasjoner.nve.no/rapport/2017/rapport2017_15.pdf.

[5] IEA, Norway - Countries & Regions, Energy mix - Norway. Accessed: Jan. 22, 2025. [Online]. Available: https://www.iea.org/countries/norway/energy-mix.

[6] IRENA, The Changing Role of Hydro Power: Challenges and Opportunities, International Renewable Energy Agency, Abu Dhabi, 2023.

[7] IEA, Hydropower Special Market Report: Analysis and Forecast to 2030. International Energy Agency, 2021, doi: 10.1787/07a7bac8-en.

[8] B.K.T. Hansen, Flood Dampening in Hydropower Systems, Master’s Thesis, NTNU, Trondheim, 2018.

[9] I. Hanssen-Bauer et al., Climate in Norway 2100 – a knowledge base for climate adaptation, Norwegian Environment Agency, Norway, NCCS report 1/2017, 2017.

[10] Sassi M. (2019). Impact of climate change on European winter and summer flood losses. Adv. Water Resour..

[11] van Vliet M.T.H. (2013). Global river discharge and water temperature under climate change. Glob. Environ. Chang..

[12] Botelho A., Ferreira P., Lima F., Pinto L.M.C., Sousa S. (2017). Assessment of the environmental impacts associated with hydropower. Renew. Sustain. Energy Rev..

[13] Moran E.F., Lopez M.C., Moore N., Müller N., Hyndman D.W. (2018). Sustainable hydropower in the 21st century. PNAS,.

[14] Hedger R.D. (2018). Modelling the effect of hydropeaking‐induced stranding mortality on Atlantic salmon population abundance. Ecohydrology.

[15] Bipa N.J., Stradiotti G., Righetti M., Pisaturo G.R. (2024). Impacts of hydropeaking: .a systematic review. Sci. Total Environ..

[16] Hayes D.S. (2024). Why hydropeaking frequency matters: effects of recurring stranding on fish. J. Ecohydraul..

[17] Berg A. (1992). Air entrainment and supersaturation of dissolved air in a shaft under atmospherical and reduced pressure conditions. J. Hydraul. Res..

[18] Huang X., Li K., Du J., Li R. (2010). Effects of gas supersaturation on lethality and avoidance responses in juvenile rock carp (Procypris rabaudi Tchang). J. Zhejiang Univ. Sci. B.

[19] Pulg U. (2024). Assessing the potential for gas supersaturation downstream of hydropower plants in Norway, Austria and Germany. Sci. Total Environ..

[20] Weitkamp D.E., Katz M. (1980). A Review of Dissolved Gas Supersaturation Literature. Trans. Am. Fish. Soc..

[21] U. Pulg et al., Gassovermetning i vassdrag - en kunnskapsoppsummering, NORCE Laboratorium for ferskvannsøkologi og innlandsfiske, Bergen, Norway, LFI rapport nr. 312, 2018.

[22] Rucker R.R. (1975). Gas bubble disease: mortalities of coho salmon, Oncorhynchus kisutch, in water with constant total gas pressure and different oxygen-nitrogen ratios. Fish. Bull..

[23] Bouck G.R. (1980). Etiology of gas bubble disease. Trans. Am. Fish. Soc..

[24] Velle G., Isaksen T.E., Lennox R.J., Pulg U. (2024). Bubbling trouble: effects of supersaturated water on benthic macroinvertebrates. Ecohydrology.

[25] Stenberg S.K., Velle G., Pulg U., Skoglund H. (2020). Acute effects of gas supersaturation on Atlantic salmon smolt in two Norwegian rivers. Hydrobiologia.

[26] Li P., Zhu D.Z., Li R., Wang Y., Crossman J.A., Kuhn W.L. (2022). Production of total dissolved gas supersaturation at hydropower facilities and its transport: a review. Water Res..

[27] Zhang B., Fu X., Li K., Li R., Guo X. (2023). Generation and release mechanism and abatement measures for gas supersaturation downstream of hydropower dams: a review. Water Resour. Res..

[28] Innovation Norway, How Norway produces hydropower with a minimal carbon footprint, Business Norway, 20, 2023. Accessed: Oct. 04, 2023. [Online]. Available: https://businessnorway.com/articles/how-norway-produces-hydropower-with-a-minimal-carbon-footprint.

[29] J.R. Fair, H.Z. Kister, Absorption (chemical engineering), in: Encyclopedia of Physical Science and Technology, Elsevier, 2003, pp. 1–25, doi: 10.1016/B0-12-227410-5/00001-6.

[30] P.M. Doran, Mass transfer, in: Bioprocess Engineering Principles, Elsevier, 2013, pp. 379–444, doi: 10.1016/B978-0-12-220851-5.00010-1.

[31] Pflieger R., Audiger G., Nikitenko S.I., Ashokkumar M. (2021). Impact of bubble coalescence in the determination of bubble sizes using a pulsed US technique: part 2 – Effect of the nature of saturating gas. Ultrason. Sonochem..

[32] Petrier C. (1994). Sonochemical degradation of phenol in dilute aqueous solutions: comparison of the reaction rates at 20 and 487 kHz. J. Phys. Chem..

[33] Eskin D.G. (2015). Power Ultrasonics.

[34] Abramov V.O., Abramova A.V., Bayazitov V.M., Nikonov R.V., Cravotto G. (2021). Pores-free aluminium alloy by efficient degassing ultrasonic treatments. Appl. Acoust..

[35] Eskin D.G., Gallego-Juárez J.A., Graff K.F., Lucas M. (2023). Woodhead Publishing Series in Electronic and Optical Materials.

[36] Asakura Y., Yasuda K. (2022). Frequency and power dependence of ultrasonic degassing. Ultrason. Sonochem..

[37] Yanagida H. (2008). The effect of dissolve gas concentration in the initial growth stage of multi cavitation bubbles: differences between vacuum degassing and ultrasound degassing. Ultrason. Sonochem..

[38] Naidji B., Hallez L., Taouil A.E., Rebetez M., Hihn J.-Y. (2019). Influence of pressure on ultrasonic cavitation activity in room temperature ionic liquids: an electrochemical study. Ultrason. Sonochem..

[39] Leong T., Ashokkumar M., Kentish S. (2011). The fundamentals of power ultrasound – a review. Acoust. Aust..

[40] E.A. Neppiras, Acoustic Cavitation, North-Holland Publishing Company, PHYSICS REPORT 3, 1980.

[41] Nomura H., Koda S. (2015). Sonochemistry and the Acoustic Bubble.

[42] Ellens N.P.K., Hynynen K. (2015). Power Ultrasonics.

[43] Gallego-Juárez J.A., Graff K.F. (2015). Power Ultrasonics.

[44] Pollet B.G., Ashokkumar M. (2019). SpringerBriefs in Molecular Science.

[45] Mason T.J. (1999).

[46] Mettin R., Cairós C. (2016). Handbook of Ultrasonics and Sonochemistry.

[47] Yasui K. (2015). Sonochemistry and the Acoustic Bubble.

[48] Lauterborn W., Mettin R. (2015). Power Ultrasonics.

[49] Eller A., Flynn H.G. (1965). Rectified diffusion during nonlinear pulsations of cavitation bubbles. J. Acoust. Soc. Am..

[50] Louisnard O., Gomez F. (2003). Growth by rectified diffusion of strongly acoustically forced gas bubbles in nearly saturated liquids. Phys. Rev. E.

[51] Louisnard O. (2017). A viable method to predict acoustic streaming in presence of cavitation. Ultrason. Sonochem..

[52] O. Louisnard, Bubble dynamics and cavitation, in: Presented at the International School in Sonochemistry, Institut de Chimie Séparative de Marcoule, France, 2023.

[53] Leighton T.G. (1994).

[54] Ye L., Zhu X., Liu Y. (2019). Numerical study on dual-frequency ultrasonic enhancing cavitation effect based on bubble dynamic evolution. Ultrason. Sonochem..

[55] Yasui K. (2002). Influence of ultrasonic frequency on multibubble sonoluminescence. J. Acoust. Soc. Am..

[56] Kumar A., Gogate P.R., Pandit A.B., Delmas H., Wilhelm A.M. (2004). Gas-liquid mass transfer studies in sonochemical reactors. Ind. Eng. Chem. Res..

[57] Yasui K., Tuziuti T., Lee J., Kozuka T., Towata A., Iida Y. (2008). The range of ambient radius for an active bubble in sonoluminescence and sonochemical reactions. J. Chem. Phys..

[58] Gondrexon N., Renaudin V., Boldo P., Gonthier Y., Bernis A., Pettier C. (1997). Degassing effect and gas-liquid transfer in a high frequency sonochemical reactor. Chem. Eng. J..

[59] Hihn J.-Y., Doche M.-L., Mandroyan A., Hallez L., Pollet B.G. (2011). Respective contribution of cavitation and convective flow to local stirring in sonoreactors. Ultrason. Sonochem..

[60] Liu L., Yang Y., Liu P., Tan W. (2014). The influence of air content in water on ultrasonic cavitation field. Ultrason. Sonochem..

[61] O.A. Kapustina, Degassing of liquids, in: Physical Principles of Ultrasonic Technology, L. D. Rozenberg, Ed., Boston, MA: Springer US, 1973, pp. 378–509, doi: 10.1007/978-1-4684-8217-1.

[62] Laugier F., Andriantsiferana C., Wilhelm A.M., Delmas H. (2008). Ultrasound in gas–liquid systems: effects on solubility and mass transfer. Ultrason. Sonochem..

[63] D.G. Eskin, Overview of Ultrasonic Degassing Development, in: Light Metals 2017, A. P. Ratvik, Ed., in: The Minerals, Metals & Materials Series, Cham: Springer International Publishing, 2017, pp. 1437–1443, doi: 10.1007/978-3-319-51541-0_171.

[64] Ou Y., Li R., Tuo Y., Niu J., Feng J., Pu X. (2016). The promotion effect of aeration on the dissipation of supersaturated total dissolved gas. Ecol. Eng..

[65] Ou Y., Li Z., Li R., Feng J., Faisal S. (2023). Experimental study on the dissipation performance of supersaturated total dissolved gas in microbubble treatment. Water Sci. Technol..

[66] Yao Y., Yang H., Wang Y. (2023). Experimental investigation and modeling on the supersaturated total dissolved gas (TDG) dissipation in aeration. Water Sci. Technol..

[67] Zhang Y., Zhou G., Prosperetti A. (2017). Bubbles as a means for the deaeration of water bodies. J. Environ. Eng..

[68] M. A. Lichtwardt, A. Murphy, Microbubble Treatment of Gas Supersaturated Water, Water Treatment Engineering and Research Group, U.S. Department of Interior, Denver, Colorado, USA, Desalination and Water Purification R&D Program Report No. 70, 2001.

[69] Žák A., Zedníková M., Moucha T. (2023). Local volumetric mass transfer coefficients in sections of multiple-impeller stirred tank reactors: data analysis. Chem. Eng. Res. Des..

[70] Shen X., Liu S., Li R., Ou Y. (2014). Experimental study on the impact of temperature on the dissipation process of supersaturated total dissolved gas. J. Environ. Sci..

[71] Feng J., Li R., Ma Q., Wang L. (2014). Experimental and field study on dissipation coefficient of supersaturated total dissolved gas. J. Cent. South Univ..

[72] Li R., Hodges B.R., Feng J., Yong X. (2013). Comparison of Supersaturated Total Dissolved Gas Dissipation with Dissolved Oxygen Dissipation and Reaeration. J. Environ. Eng.,.

[73] Huang J., Li R., Feng J., Xu W., Wang L. (2016). Relationship investigation between the dissipation process of supersaturated total dissolved gas and wind effect. Ecol. Eng..

[74] Fickeisen D.H., Schneider M.J., Schneider M.J., Montgomery J.C., Montgomery J.C. (1975). A comparative evaluation of the Weiss Saturometer. Trans. Am. Fish. Soc..

[75] Pleizier N., Cooke S.J., Brauner C.J. (2021). A simple chamber design for calibrating Weiss Saturometers and recommendations for measuring and reporting total dissolved gases. Water Resour. Res..

[76] D’Aoust B.G. (2007). Technical note: total dissolved gas pressure (TDGP) sensing in the laboratory. Dissolution Technol..

[77] Margulis M.A., Margulis I.M. (2003). Calorimetric method for measurement of acoustic power absorbed in a volume of a liquid. Ultrason. Sonochem..

[78] Hansen H.E., Seland F., Sunde S., Burheim O.S., Pollet B.G. (2021). Two routes for sonochemical synthesis of platinum nanoparticles with narrow size distribution. Mater. Adv.,.

[79] Carr J.J. (1993).

[80] Agarkoti C., Gogate P.R. (2022). Mapping of cavitation intensity in a novel dual-frequency ultrasonic reactor of capacity 10 L. Chem. Eng. Sci..

[81] Pincovschi I., Dragomirescu A., Modrogan C. (2025). Influence of impeller design on oxygen transfer in a stirred water tank with square cross-section. Biochem. Eng. J..

[82] Wang Z. (2020). Effects of bubble size on the gas–liquid mass transfer of bubble swarms with Sauter mean diameters of 0.38–4.88 mm in a co‐current upflow bubble column. J. Chem. Technol. Biotechnol..

[83] Motarjemi M., Jameson G.J. (1978). Mass transfer from very small bubbles—the optimum bubble size for aeration. Chem. Eng. Sci..

[84] Ham P., Bun S., Painmanakul P., Wongwailikhit K. (2021). Effective analysis of different gas diffusers on bubble hydrodynamics in bubble column and airlift reactors towards mass transfer enhancement. Processes.

[85] Sharma H., Nirmalkar N. (2022). Enhanced gas-liquid mass transfer coefficient by bulk nanobubbles in water. Mater. Today Proc..

[86] Barthès M., Mazue G., Bonnet D., Viennet R., Hihn J.-Y., Bailly Y. (2015). Characterization of the activity of ultrasound emitted in a perpendicular liquid flow using Particle Image Velocimetry (PIV) and electrochemical mass transfer measurements. Ultrasonics.

[87] Mazue G. (2015). Influence of a perpendicular liquid flow on a cleaning process using 20 kHz ultrasound: characterization of the agitation at vicinity of the surface opposite to the transducer. Can. J. Chem. Eng..

[88] Sajjadi B., Asgharzadehahmadi S., Asaithambi P., Raman A.A.A., Parthasarathy R. (2017). Investigation of mass transfer intensification under power ultrasound irradiation using 3D computational simulation: a comparative analysis. Ultrason. Sonochem..

[89] Kuhn W.L., Solemslie B.W., Hihn J.-Y., Dahlhaug O.G. (2023). Evaluating natural degassing in a river to create a baseline for comparison to technical degassing methods. J. Phys. Conf. Ser.,.

